# Interplay among Gcn5, Sch9 and Mitochondria during Chronological Aging of Wine Yeast Is Dependent on Growth Conditions

**DOI:** 10.1371/journal.pone.0117267

**Published:** 2015-02-06

**Authors:** Cecilia Picazo, Helena Orozco, Emilia Matallana, Agustín Aranda

**Affiliations:** 1 Department of Biotechnology, Institute of Agrochemistry and Food Technology, IATA-CSIC, Paterna, Spain; 2 Departament of Biochemistry and Molecular Biology, University of Valencia, Burjassot, Spain; Duke University Medical Center, UNITED STATES

## Abstract

*Saccharomyces cerevisiae* chronological life span (CLS) is determined by a wide variety of environmental and genetic factors. Nutrient limitation without malnutrition, i.e. dietary restriction, expands CLS through the control of nutrient signaling pathways, of which TOR/Sch9 has proven to be the most relevant, particularly under nitrogen deprivation. The use of prototrophic wine yeast allows a better understanding of the role of nitrogen in longevity in natural and more demanding environments, such as grape juice fermentation. We previously showed that acetyltransferase Gcn5, a member of the SAGA complex, has opposite effects on CLS under laboratory and winemaking conditions, and is detrimental under the latter. Here we demonstrate that integrity of the SAGA complex is necessary for prolonged longevity, as its dismantling by *SPT20* deletion causes a drop in CLS under both laboratory and winemaking conditions. The *sch9*Δ mutant is long-lived in synthetic SC medium, as expected, and the combined deletion of *GCN5* partially suppresses this phenotype. However it is short-lived in grape juice, likely due to its low nitrogen/carbon ratio. Therefore, unbalance of nutrients can be more relevant for life span than total amounts of them. Deletion of *RTG2*, which codes for a protein associated with Gcn5 and is a component of the mitochondrial retrograde signal, and which communicates mitochondrial dysfunction to the nucleus, is detrimental under laboratory, but not under winemaking conditions, where respiration seems not so relevant for longevity. Transcription factor Rgm1 was found to be a novel CLS regulator Sch9-dependently.

## Introduction

The yeast *Saccharomyces cerevisiae* is a very useful biotechnological tool thanks to its ability to perform alcoholic fermentation, the metabolic process underlying baking, brewing, winemaking and bioethanol production. However, industrial strains show some genetic differences to the strains used in laboratories [1]. Commercial wine yeasts are prototrophs and can produce all their amino acids from a single nitrogen source [2], while laboratory strains are generally mutants in the genes involved in amino acid or nitrogen-base biosynthesis. Overall, industrial yeast strains are more robust and more stress-tolerant to the environmental challenges they face during winemaking, particularly initial high sugar concentration (around 20%), low nitrogen and oxygen levels, and high final ethanol content [3].

*S. cerevisiae* has been widely used as a eukaryotic model for studying the molecular mechanisms that modulate life span given their high conservation from yeast to mammals [4,5]. In yeast two models of aging, replicative life span (RLS) and chronological life span (CLS), occur. RLS is defined as the number of daughter cells produced by a mother cell, whereas CLS is defined as the capacity of stationary cells to maintain viability in a nondividing state. CLS is the longevity model of postmitotic cells that constitute bulk of tissue in mammals. From the industrial point of view, studying chronological longevity is relevant when a yeast culture no longer divides, as occurs at the end of alcoholic fermentation. Accumulation of damaged proteins and mitochondria with time can cause cell death in both aging types [4], and metabolites, such as ethanol and acetic acid, have been shown to be pro-aging factors in chronological aging

Various regulatory mechanisms are important for determining longevity, including nutrient signaling pathways, acetylation/deacetylation machinery (mainly sirtuins), stress responses and autophagy. Nutrient signaling pathways regulate cell growth and proliferation, metabolism and stress responses. They allow cells to not only stimulate metabolism and growth when nutrients are present, but to also enter the stationary phase during nutrient starvation periods, thus improving long-term survival. The main environmental alteration that extends longevity is decreased nutrient supply without inducing malnutrition, which is called dietary restriction. In yeast, this can occur by reducing the intake of nitrogen or carbon sources, and involves the Ras/cAMP/PKA and TOR/Sch9 pathways [6]. TOR (Target Of Rapamycin), and its related kinase Sch9, control cell growth and metabolism in response to nutrients, which highlights the response to nitrogen availability. The use of chemical inhibitors of TOR, such as rapamycin, or mutations in TOR/Sch9 pathway proteins causes diminished pathway activity that promotes an extension of yeast longevity [7,8]. We previously found that the chemical inhibition of TOR extends CLS under winemaking conditions [9]. In starvation, *S. cerevisiae* induces autophagy, a highly conserved catabolic process in eukaryotes, which allows the recycling of intracellular components by degradation in the lytic compartment (vacuoles in yeast) [10]. Thus, the cell may obtain nutrients to allow survival in nutritional shortage. However, excessive autophagy can lead to cell death, so it is necessary to keep the process within a physiological range [11]. For instance, in grape juice fermentation autophagy promotes chronological aging [9].

Environmental conditions are also a key factor for onset of CLS. Winemaking fermentation conditions by industrial *S. cerevisiae* strains vastly differ from standard laboratory environments. Grape juice is very rich in sugars (up to 20–25%), but poor in nitrogen sources [12]. Therefore, cell division arrest occurs when the carbon source is plentiful. In fact most sugar consumption and ethanol production happens when cells are nondividing or dying, so this is a very interesting process from the biotechnological viewpoint. Cell death also happens under high ethanol and low oxygen conditions, so different rules may apply to molecular aging mechanisms. In previous works, we have shown that the mutation of acetyltransferase Gcn5 inhibits autophagy under laboratory and industrial conditions [9]. It plays a positive role in lifespan under standard laboratory conditions of growth in synthetic complete (SC) medium, but its deletion extends CLS under winemaking conditions, suggesting that physiological conditions affect the way that some mechanisms, such autophagy, control aging. Ethanol production in the stationary phase is a key factor for CLS under both laboratory and industrial conditions [13,14]. Gcn5 forms part of the SAGA (Spt-Ada-Gcn5 acetyltransferase) complex, involved in gene expression, from the start of transcription to mRNA transport. The SAGA complex is composed of four modules, two of them with enzymatic activity, the acetyltransferase module (HAT) and the deubiquitinylase (DUB) module (the latter interacts with RNA polymerase II), the TAF module, and a structural module, SPT [15]. Strains lacking genes encoding the DUB module (*SGF73*, *SGF11* and *UBP8*) have an extended replicative life span [16]. That is not the case for other SAGA components, like *GCN5*. Acetyltransferase Gcn5 is also associated with another protein complex, SLIK (SAGA-like) [17], which includes Rtg2, a component not found in SAGA. Rtg2 is a central component of the yeast retrograde response pathway [18], which allows communication between mitochondria and the nucleus for the response to mitochondrial stress [19]. The retrograde response lies at the nexus of metabolic regulation, stress resistance, chromatin-dependent gene regulation and aging. Rtg2 is required for the replicative life span extension caused by mitochondrial malfunction [20]. Gcn5 modulates the retrograde response as deletion of *GCN5* prevents increased replicative longevity caused by induction of the retrograde response [21].

We herein analyzed the role of the proteins that may interact with acetyltransferase Gcn5 on lifespan regulation under standard laboratory conditions and during winemaking, and both physical and genetic interactions were identified. We found that the integrity of the SAGA complex is relevant for longevity and autophagy. We further investigated the relationship between both Gcn5 and the Sch9 kinase and the retrograde response in chronological aging. Gcn5 is relevant for the CLS extension caused by Sch9 deletion, so it may orchestrate the gene expression pattern set by the kinase. The role of Sch9 under winemaking conditions is to promote life span, probably due to nitrogen starvation conditions.

## Materials and Methods

### Yeast strains and growth media

S1 Table lists the industrial wine yeasts used in this work. Haploid strain C9 (*Mat* a, *ho*::*loxP*) was a gift from Michelle Walker [22]. Industrial wine yeast L2056 was kindly provided by Lallemand Inc. (Montreal, Canada). Gene disruptions were performed by using recyclable selection marker *loxP*-*kanMX*-*loxP* from plasmid pUG6 [23]. The marker was eliminated by transforming with cre recombinase-containing plasmid YEp351-cre-cyh [24]. S2 Table lists the oligonucleotides employed to amplify deletion cassettes and to check transformants. The *petite* strains were obtained by growth in SD medium with ethidium bromide (10 μg/mL), where yeast lost the functional mitochondria and became a strictly aerobic *petite* strain, uncapable of growth in nonfermentable carbon sources (e.g., glycerol)[25].

For yeast growth, YPD medium (1% yeast extract, 2% bactopeptone, 2% glucose) was used. SC medium contained 0.17% yeast nitrogen base, 0.5% ammonium sulfate, 2% glucose and 0.2% drop-out mix with all the amino acids [26]. SD-N is as SC with no ammonium sulfate and amino acids. SC N 1/25 is like SC, with 25-fold less ammonium sulfate and amino acids. Solid plates contained 2% agar and 20 μg mL^-1^ geneticin or 0.1 μg mL^-1^ cycloheximide. Red grape juice (Tempranillo variety) was a gift from Bodegas J. Belda (Fontanars dels Alforins, Spain). It was sterilized overnight with 500 μg/L of dimethyl dicarbonate.

### Yeast growth conditions and chronological life span measurements

For the CLS experiments done under laboratory conditions, precultures of selected strains were grown overnight on YPD and were then inoculated in SC media at an OD_600_ of 0.1. After 3 days of growth at 30°C, aliquots were taken, diluted and plated. Colonies were counted and the percentage of survival was calculated by taking day 3 of growth as 100% survival.

For the microvinification experiments, the cells from 2-day cultures in YPD were inoculated at a final concentration of 10^6^ cells/mL in filled-in conical centrifuge tubes with 30 mL of grape juice. Incubation was done with very low shaking at 24°C. Vinification progress was followed by determining cell viability and sugar consumption, as previously described [27]. Survival plots were drawn by taking the highest cell viability point (around 2–5 days) as 100% survival.

### Metabolite determinations and Western blotting

Reducing sugars during fermentation were measured by the reaction to DNS (dinitro-3,5-salycilic acid)[28]. Ethanol was measured with the kits provided by r-Biopharm following the manufacturer’s instructions.

For the autophagy measurements, Ald6 levels were detected by Western blot [29]. At different growth times in SD-N medium, cells were taken and broken with one volume of glass beads in a buffer containing Tris-HCl 0.1 M pH 7.5, NaCl 0.5 M, MgCl_2_ 0.1 M, NP40 1% (v/v), PMSF 10 mM and protease inhibitors (complete Mini, EDTA-free from Roche). Protein concentration was measured by the Bradford method using the Bio-Rad Protein assay following the manufacturer’s instructions. Extracts were diluted in loading buffer for SDS-PAGE (Tris-HCl 240 mM pH 6.8, SDS 8% (p/v), glycerol 40%, β-mercaptoethanol 10%). After electrophoresis, the gel was blotted onto PVDF membranes for the Western blot analysis with an Invitrogen mini-gel device. The anti-ALDH antibody was obtained from Rockland (Gilberstville, USA) and the anti-ADH antibody was obtained from Acris (Hiddenhausen, Germany). The ECL Western blotting detection system (Amersham) was used following the manufacturer´s instructions.

## Results

### SAGA complex components Ubp8 and Spt20 regulate longevity and autophagy

**Fig 1:**
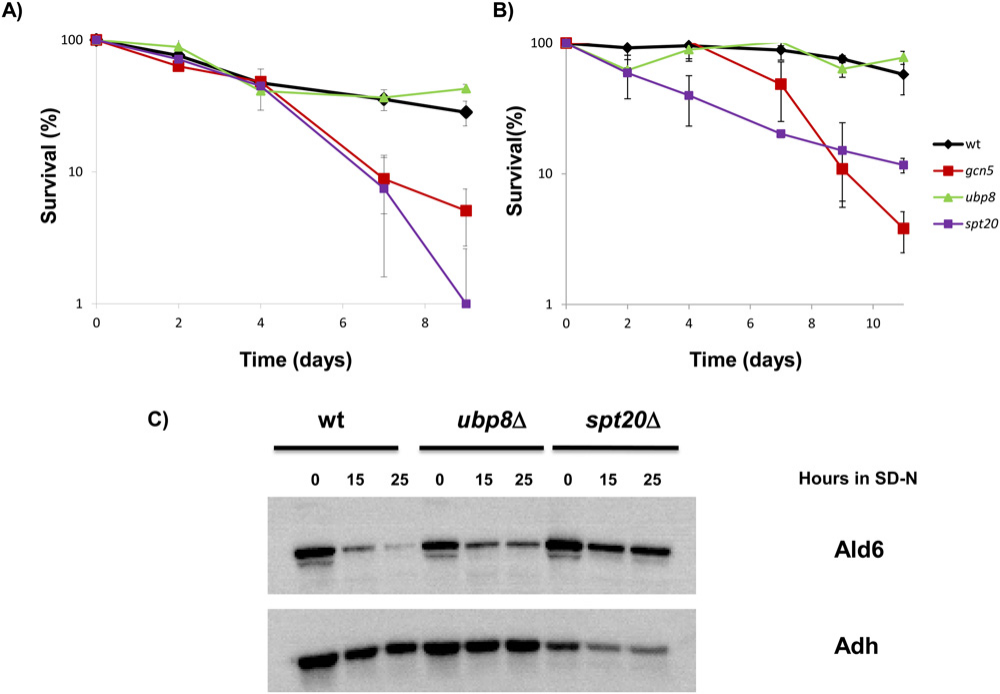
Spt20 plays a role in life span and autophagy control. **A)** Survival curves in minimal complete medium SC for the SAGA complex mutants in wine strain C9. Cell number at day 3 after inoculation was considered to be 100% viability. All the experiments were carried out in triplicate and the mean and standard deviation are shown. **B)** Survival curves in low nitrogen (SC N 1/25) medium for the same mutants. Cell numbers at day 7 were considered to be 100% viability. **C)** Western blot detection of Ald6 in the wild type and the *ubp8*Δ and *spt20*Δ mutants in minimal medium with no nitrogen source (SD-N). Alcohol dehydrogenase (Adh) was used as the loading control.

In a previous work, we demonstrated the role of acetyltransferase Gcn5 in autophagy and in CLS control [9]. In order to study the role of other SAGA complex members in those processes, we proceeded to generate deletion mutants for the genes coding for SAGA proteins in the C9 wine strain to analyze the chronological aging profile in their absence. The C9 strain is a haploid derivative of the diploid L2056 commercial wine yeast strain [22], where the construction of single and double mutants is easier, thus it was used throughout this work. We deleted the *UBP8* gene that codes for the deubiquitinylation activity of the complex and the *SPT20* gene, which codes for the structural Spt20 component, whose absence dismantles the SAGA complex [30]. First, the chronological aging of these strains was studied in standard laboratory minimal complete medium (SC). The wild-type strain and the *ubp8*Δ mutant have a similar chronological aging profile (Fig. 1A), suggesting that the deubiquitinylase activity of SAGA is not relevant for longevity. However, the *spt20*Δ mutant presents shorter maximum CLS than the parental strain, which is a similar result to that observed for the *gcn5*Δ mutant. Therefore, the enzymatic activity of Gcn5 that is involved in longevity seems to occur in the context of SAGA. In order to study the relevance of these genes in a dietary restriction context caused by nitrogen depletion, a similar medium with 25-fold less nitrogen was tested (SC 1/25N), which mimics the low nitrogen conditions of grape musts for winemaking (Fig. 1B). In this case, growth was slower, so the time point corresponding to day 7 was taken as 100% survival instead of day 3. Life span was extended with a low nitrogen concentration for the wild-type strain when compared to the rich medium, as expected under the dietary restriction condition, and the mean life span (50% viability) was extended by 2.5-fold (S3 Table). Once again, the *UBP8* deletion mutant had a similar profile to the parental strain (Fig. 1B), which reinforces the conclusion that this activity of the SAGA complex does not seem to play a relevant role in longevity under high or low nitrogen conditions. The mean life span was extended in the *gcn5*Δ mutant when compared to SC medium, but this extension was not observed in the *spt20*Δ mutant, which even reduced it (from 3.5 days to 3 days; S3 Table). Therefore, the integrity of the SAGA complex may play a role in the extension of longevity caused by the nitrogen dietary restriction. Thus Gcn5 and Spt20 are relevant for longevity, independently of the nitrogen content of the medium as the strains deleted for these proteins have shorter maximum life spans in any media when compared to the parental strain.

As mentioned in the Introduction, acetyltransferase Gcn5 controls autophagy in wine yeast [9], as demonstrated by the stabilization of the levels of cytosolic aldehyde dehydrogenase Ald6, a selective marker of autophagy in response to nitrogen starvation [31]. Experiments to analyze autophagy in mutants *ubp8*Δ and *spt20*Δ (Fig. 1C) were conducted by the Western Blot detection of Ald6 and by using a C9 strain, where the gene coding for the mitochondrial *ALD4* was deleted to prevent cross-detection. Alcohol dehydrogenase (Adh) was used as loading control. This protein is not degraded by autophagy [31], but interstingly its basal levels are decreased in the *spt20*Δ mutant. The *ubp8*Δ mutant presents a small defect in autophagy, with slightly higher Ald6 levels than the wild-type strain. The results for the *spt20*Δ mutant indicate a more marked defect in autophagy, with high Ald6 levels after nitrogen depletion. This indicates that the integrity of the SAGA complex is important for autophagy, and that its loss might participate in the shortening of CLS in this mutant under laboratory conditions.

**Fig 2:**
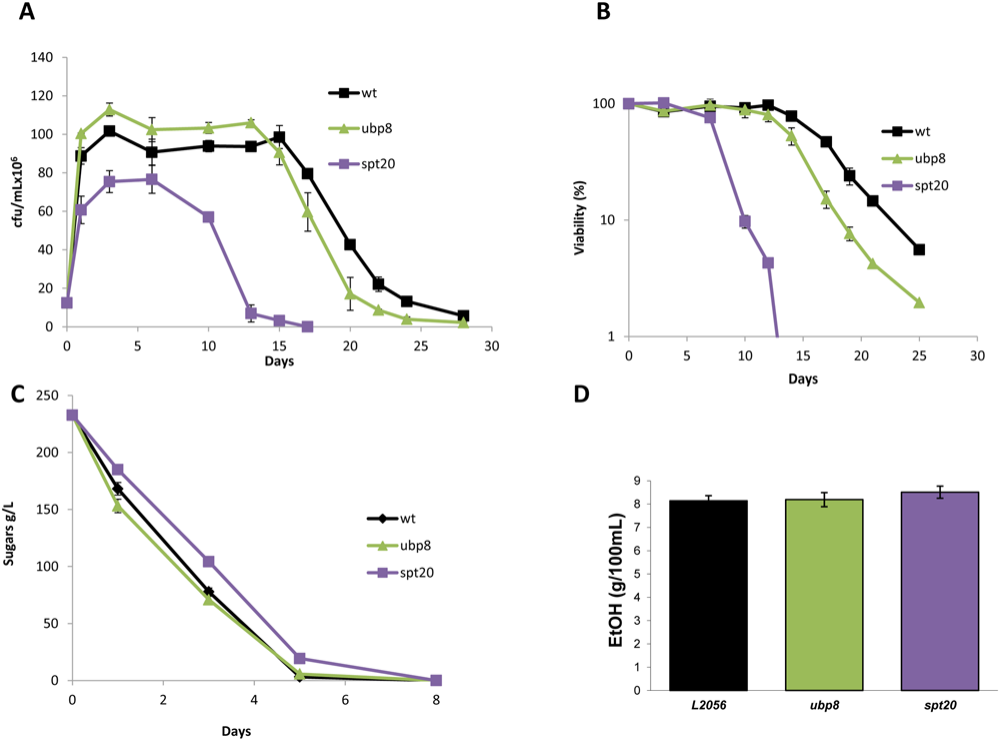
Spt20 plays a role in growth and aging under winemaking conditions. **A)** Growth curves for wine strain L2056 and its derivatives *spt20*Δ and *ubp8*Δ showing the number of viable cells (cfu/mL) determined by plate counting at different times during winemaking in natural grape juice. Experiments were performed at least in triplicate, and errors bars show the standard deviation (SD). **B)** Survival curves for the same strains. The cell numbers at day 3 in panel A were taken as 100% viability. **C)** Sugar consumption profiles during fermentation. **D)** Ethanol production at the end of grape juice fermentation. Ethanol was measured when sugars were completely consumed (below 2 g/l).

Next, in order to analyze the effect of these mutations under winemaking conditions, the two copies of *UBP8* and *SPT20* genes were deleted from commercial wine strain L2056 and the mutants were grown in a natural medium, red grape juice. Cell growth during fermentation is shown in Fig. 2A. The *ubp8*Δ mutant growth profile is similar to the wild type and no significant differences in maximum cell growth were found, although viability dropped faster in the last stages. Mutation of the *SPT20* gene led to a dramatic change in the growth profile, with significantly reduced total cell growth and accelerated loss of viability. Slower growth in YPD rich medium was also observed (data not shown), which indicates that poor growth of the mutant is not dependent on the media. Cell viability at day 3, where cell density of the *spt20*Δ mutant peaked, was taken as 100% survival to plot the CLS curves of these strains (Fig. 2B). The CLS of *ubp8*Δ strain was shorter if compared to the parental strain. Therefore, Ubp8 has an impact on life span in natural media, which contrasts with the absence of effects observed in the laboratory media. Once again, the *spt20*Δ mutant displayed a more markedly reduced CLS as compared to the wild-type strains, which also happened in SC medium. These results demonstrate that SAGA integrity is relevant to achieve full CLS under different environmental conditions, whereas the effects of the deubiquitynilation activity of Ubp8 are growth medium-dependent.

To follow the evolution of vinification and to determine the impact of these mutations on the ability to complete wine fermentation, samples were taken at different times during fermentative growth and the level of reducing sugars (glucose and fructose) were determined (Fig. 2C). The sugar consumption of the *spt20*Δ mutant was lower than consumption of the parental strain, probably due to the low maximum cell number and the viability lost in this mutant. The *ubp8*Δ mutant, however, showed a similar consumption rate to the parental strain, which is consistent with the similar maximum cell number and viability during the fermentation period, and indicates that there the deletion of this gene had no major impact on global metabolism. The impact that the lack of these proteins in ethanol production (a well-known pro-aging metabolite [13]) was also determined. Fig. 2D shows that no mutant led to significant differences in the final amount of ethanol, so overall fermentative metabolism was not challenged and differences in life spans were not due to differences in ethanol concentration.

The experiments performed with the mutants constructed in commercial wine strain L2056 were repeated for their equivalent in the haploid C9 strain, and both *ubp8*Δ and *spt20*Δ gave the same results, with deletion of *SPT20* causing lower maximum cell density (S1A Fig.). Both deletions also caused a shorter maximum CLS (S1B Fig.) and no differences in ethanol production were observed (S1D Fig.). Once more, *spt20*Δ showed slower sugar assimilation (S1C Fig.). Therefore as similar results were obtained in both genetic backgrounds, the C9 strain was selected to perform the remaining experiments for simplicity.

### Role of the retrograde response in chronological aging

**Fig 3:**
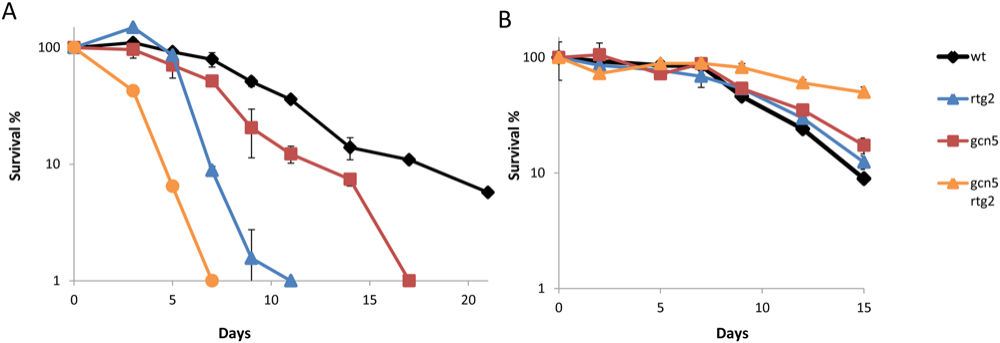
Deletions of *RTG2* and *GCN5* have additive effects. **A)** Survival curves of mutants *rtg2*Δ and *gcn5*Δ in SC medium. Conditions as in Fig. 1A. **B)** Survival plots during natural grape juice fermentation. Conditions as in Fig. 2B. Experiments were performed in triplicate. Error bars show the standard deviation (SD).

SLIK is a complex similar to SAGA and contains Gcn5, Ubp8 and Spt20, but also includes the Rtg2 protein. Rtg2 belongs to the retrograde response pathway, which informs the nucleus of mitochondrial status. For this reason, we decided to study the relationship between both the *RTG2* and *GCN5* genes in the chronological life span context in wine yeast under different environmental conditions (Fig. 3). First, the simple *rtg2*Δ and *gcn5*Δ mutations, and the double *rtg2*Δ *gcn5*Δ mutant, were tested for CLS in laboratory medium SC (Fig. 3A). Deletion of *RTG2* causes sharp reduction in CLS if compared to the wild-type strain, and the effect was much stronger than for the *gcn5*Δ mutant. Therefore, the retrograde response is necessary to achieve full life span during long-term growth in SC, when respiratory metabolism occurs. Lifespan reduced even more when both mutations were combined in the double mutant, indicating that deleterious effects of both mutations take place, at least partially, through independent pathways. Next the behavior of these mutants was tested during grape juice fermentation and, in this case (Fig. 3B), deletion of *RTG2* slightly extended life span and, as previously described, also *GCN5* deletion. The double *rtg2*Δ *gcn5*Δ mutant further extended CLS. Therefore, the effect of these two mutations, be it contrary under the two different growth conditions, is also additive and suggests two independent pathways, but both seemed to similarly react to changes in the environment by increasing or decreasing life span, depending on the medium. The *RTG2* and *GCN5* mutations did not cause relevant defects in fermentative metabolism, as reflected by the similar sugar consumption rate and ethanol production (S2 Fig.). Therefore, signaling from the mitochondria is detrimental for CLS when metabolism is purely fermentative, as occurs in wine fermentation.

### Interaction between Gcn5 and nutrient sensing kinase Sch9

**Fig 4:**
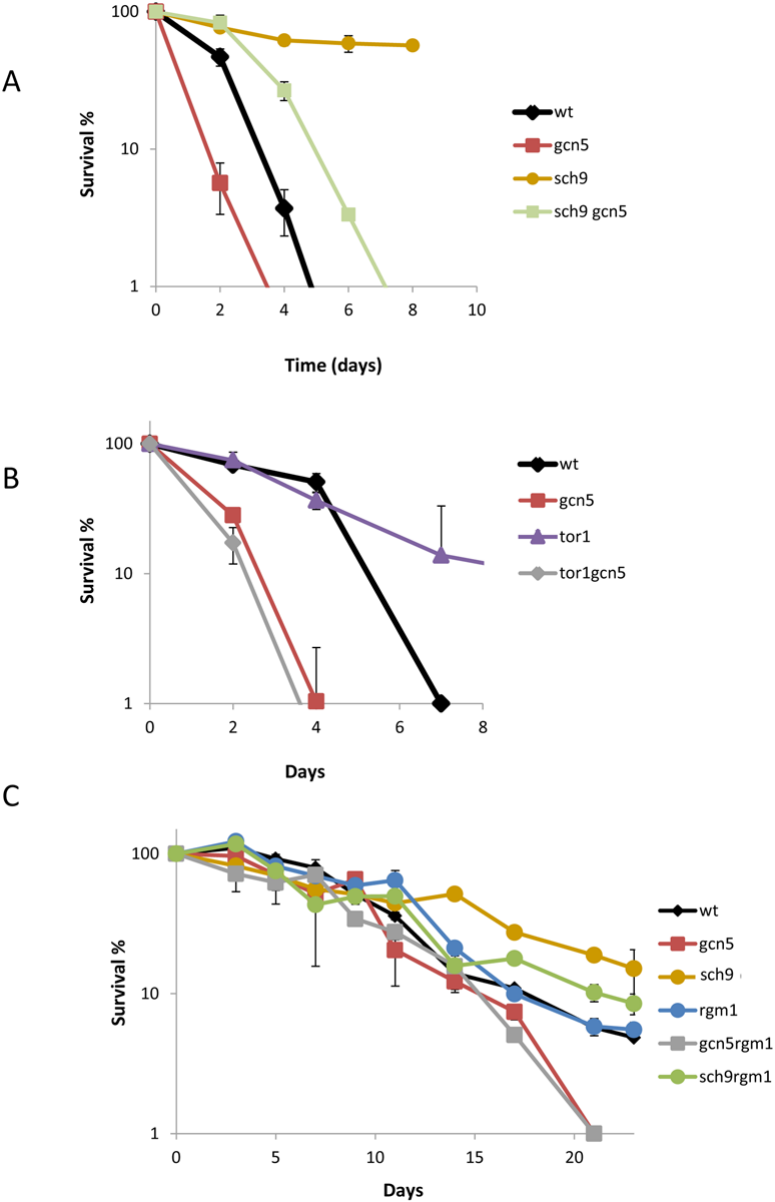
Effect of the TOR/Sch9 pathway on chronological life span in the *gcn5*Δ mutant in SC medium. **A)** Survival curves of the wild type and the *gcn5*Δ, *sch9*Δ and double mutants. The cell numbers at day 3 after inoculation were taken as 100% viability. **B)** Survival curves of the wild type and the *gcn5*Δ, *tor1*Δ and double mutants. **C)** Survival curves of the single and double mutants in genes *RGM1*, *GCN5* and *SCH9*. Experiments were performed in triplicate. Error bars show the standard deviation (SD).

*GCN5* deletion blocks the CLS extension caused by partial TOR/Sch9 inhibition during winemaking [9]. Sch9 is a key kinase in which several pathways, such as TOR, Snf1 and stress by sphingolipids [32,33] signaling, converge to control lifespan. Hence the relationship between Gcn5 and Sch9 was studied. The CLS phenotypes of the single mutants analyzed in standard SC medium were opposite; whereas the *gcn5*Δ mutant had a shortened CLS if compared to the wild type, as mentioned above (Fig. 1A), the *sch9*Δ mutant had a significantly prolonged lifespan, as expected (Fig. 4A). When both deletions took place in the double mutant, the CLS extension caused by *SCH9* deletion was partially blocked. Therefore, it seems that at least part of the mechanisms promoting longevity in the *sch9*Δ mutant requires Gcn5 activity. A similar experiment was performed with the *tor1*Δ mutant (Fig. 4B), which also displayed extended maximum life span, but behaved as the parental strain in mean CLS terms. Once again, the combined deletion of *GCN5* blocked this CLS extension, and completely so in this case, which suggests a closer functional relationship between these two proteins. Therefore, it seems clear that the expression changes and mechanisms triggered by TOR/Sch9 inhibition and producing CLS extension require the function of acetyltransferase Gcn5, at least partially.

Given the important role of Sch9 in CLS, we searched the Saccharomyces Genome Database (SGD) to find the physical and genetic connections between Gcn5 and Sch9. Sch9 interacts physically and genetically to transcription factor Rgm1 [34], which has been linked to subtellomeric binding [35]. *RGM1* also interacts genetically to *GCN5* [36], so we further investigated the role and interaction of this gene in aging. Deletion of *RGM1* did not significantly alter CLS (Fig. 4C). When combined with *GCN5* deletion, the phenotype of the double mutant was similar to the single *gcn5*Δ mutation, thus suggesting that Rgm1 plays no role in CLS regulation. Nevertheless, the CLS extension displayed by the *sch9*Δ mutant was partially blocked by the absence of Rgm1p, as observed in the double *sch9*Δ *rgm1*Δ. Therefore, Rgm1 may not play a relevant role during cell growth, but might be important for longevity in starvation, which are mimicked by the *sch9*Δ mutation.

**Fig 5:**
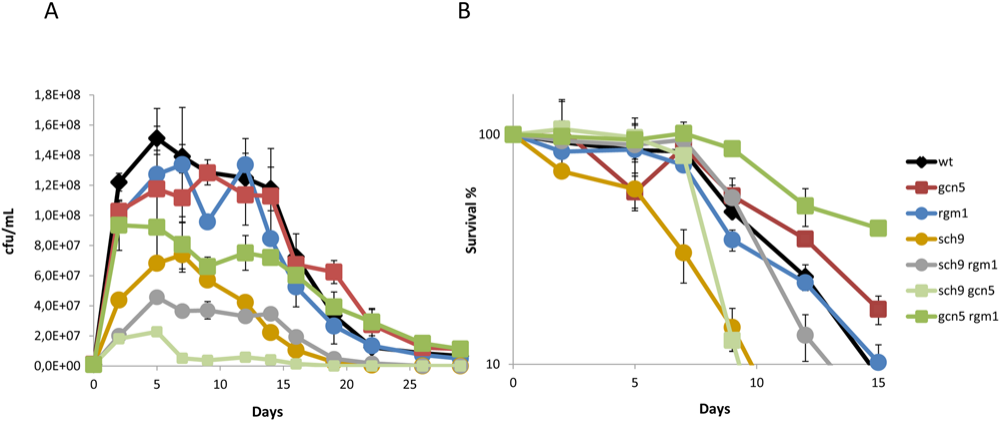
Sch9 is required for full life span under winemaking conditions. **A)** Growth curves for mutant wine yeast *sch9* Δ, *gcn5*Δ and *sch9*Δ and their combinations, during natural grape juice fermentation. The number of viable cells (cfu/mL) by plate counting at different times during winemaking evolution was determined. **B)** Survival curves considering day 7 in panel A) to be 100% viability. Experiments were performed in triplicate. Error bars show the standard deviation (SD).

The CLS phenotype of these mutants was analyzed during wine fermentation in natural grape juice (Fig. 5), where major differences in growth profiles were observed (Fig. 5A). The *sch9*Δ mutation caused a strong growth defect if compared to the wild type, and the remaining single mutants obtained very low cell densities. This fact indicates that Sch9 is relevant for growth in wine fermentations, as expected for a pathway that promotes protein synthesis. This phenotype became more striking when combining the *sch9*Δ mutation with *rgm1*Δ and particularly to *gcn5*Δ, where cell numbers were very small. The *rgm1*Δ and *gcn5*Δ single mutants had similar growth profiles, and were also similar to the parental strain, although their combination gave small cell numbers. For this experiment, 100% viability to obtain the CLS plot profile was fixed at day 7, the day when the viability of *sch9*Δ peaked (Fig. 5B). Deletion of *RGM1* led to no significant change in lifespan, as happened in SC medium, while *GCN5* deletion extended CLS under the grape juice fermentation conditions, as previously described [9]. Surprisingly, *SCH9* deletion brought about shortened CLS (both mean and maximum, see S3 Table), and the opposite effect was observed in SC (Fig. 4A). This result indicates that Sch9 inhibition does not extend life span independently of the environmental conditions, and that its function is influenced by growth conditions, as previously observed for other mutants in longevity genes under winemaking conditions [9,37]. When the *SCH9* and *GCN5* deletions were combined, maximum life span sharply reduced, which confirms the complex functional interaction between these two proteins. It is noteworthy that *RGM1* deletion was able to extend life span in the short-lived *sch9*Δ mutant and also in long-lived *gcn5*Δ, thus reinforcing the hypothesis that acetyltranferase Gcn5 plays a negative role in longevity under some environmental conditions, such as winemaking. For metabolite production (S3 Fig.), no variation in the final ethanol concentration was noted, despite the very different growth and death profiles of theses strains. In sugar consumption terms, mutants *sch9*Δ and *gcn5*Δ, and particularly the combination of both, showed a slower sugar metabolism profile, probably due to their lower cell densities, although they were all able to complete fermentation.

### Physiological cell status modulates the role of Sch9 in aging

**Fig 6:**
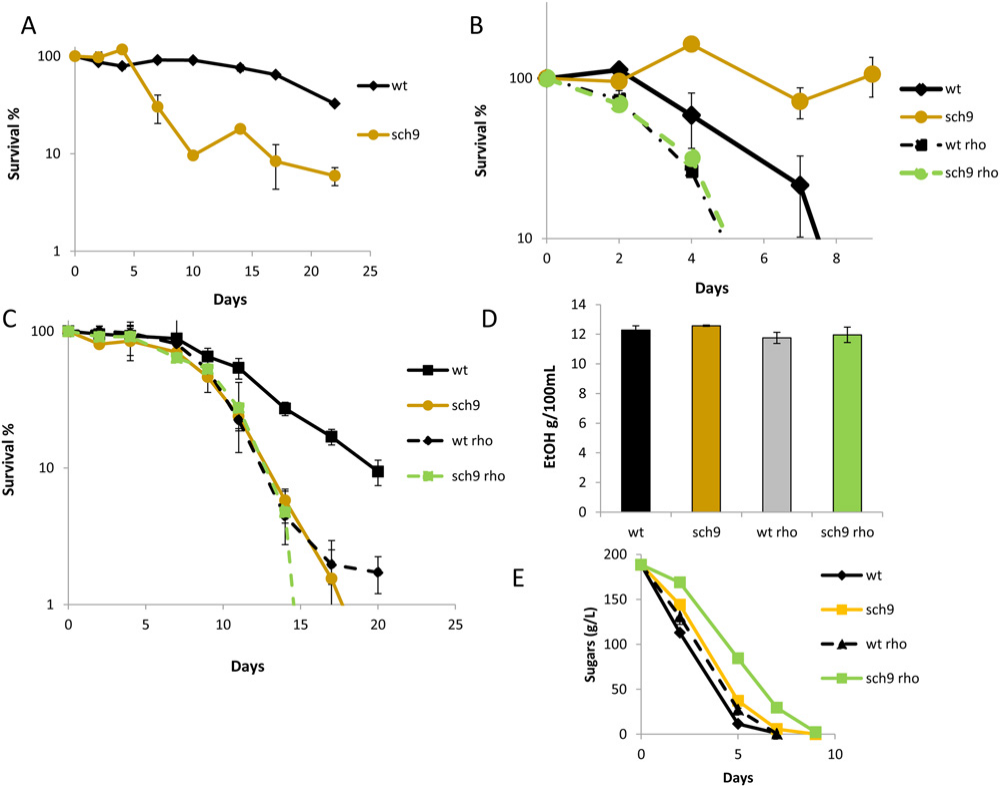
CLS extension by mitochondrial function during winemaking requires Sch9. **A)** Survival curves of the wild type and the *sch9*Δ mutant in SC containing 25-fold less nitrogen. 100% viability was taken at day 7. **B)** Survival plot of the *rho* ^*o*^ strains derived from the same strains in SC medium. 100% viability was taken at day 3. **C)** Survival plots in grape juice fermentation of the strains tested in panel B). **D)** Ethanol production during grape juice fermentation is shown in panel C). **E)** The sugar consumption profile for the aforementioned fermentation. Experiments were performed in triplicate. Error bars show the standard deviation (SD).

Given the significant and unexpected reduction of CLS during grape juice fermentation caused by *SCH9* deletion, the phenotype of the *sch9∆* mutant was further investigated. Winemaking conditions differ for growth in laboratory medium in several aspects, particularly in the low nitrogen and high sugars concentrations present in the medium, and also in the low oxygen environment that favors fermentative over-respiratory metabolism. To mimic low nitrogen, we tested the effect of *SCH9* deletion on SC medium containing 25-fold fewer amino acids and ammonium [9] (Fig. 6A). Under these conditions, the *sch9*Δ mutant was more short-lived than the parental strain, which contrasts with the CLS extension observed in standard SC medium (Fig. 4A). Therefore under the unbalanced low nitrogen and high sugar conditions present in natural grape juice and in synthetic SC 1/25 N, Sch9 is required to achieve full life span. It has been pointed out that repression of TOR/Sch9 produces life span extension by promoting respiration [38,39]. Such behavior would have little effect under the low respiration conditions of grape juice fermentation, and would explain the opposite effect of *SCH9* deletion. To gain a better understanding of the role that respiration plays under such conditions, *petite* mutants (*rho* ^o^), which are unable to grow in a nonfermentable carbon source such as glycerol, were obtained for wild-type wine strain C9 and its *sch9*Δ derivative. These strains were grown in SC to obtain the CLS profile (Fig. 6B). The C9 petite *rho* ^o^ mutant has a reduced CLS, indicating that under these conditions with plenty of aeration, mitochondria and respiration are necessary to retain full longevity. Interestingly, lack of active mitochondria has an impact on CLS during fermentation, which is the opposite result to that observed in RLS [20]: CLS is shorter in the *petite* strain compared to the *grande* normal strain. The *grande sch9*Δ mutant has an extended life span, as expected, but the *petite* version of the *sch9*Δ mutant has the same CLS as the wild-type *petite* strain. This finding therefore confirms that respiration is essential for the role of Sch9 in controlling CLS under standard laboratory conditions.

Next these *petite* strains were tested during grape juice fermentation (Fig. 6C). Under these conditions, the wild-type *rho* ^o^ mutant also had a reduced CLS. Therefore even during fermentative metabolism with a high sugar concentration, mitochondria play a positive role in longevity, which may not be related to its function in respiration. The combination of deleting the *SCH9* and *petite* mutations did not further alter CLS, which suggests a functional relationship between both factors (Fig. 6C). Regarding the metabolic status of the *petite* cells, they produced similar amounts of ethanol at the end of fermentation (Fig. 6D), and the rate of sugar consumption was slightly lower than that of the wild-type strain, although both completed fermentation at the same time (Fig. 6E). As indicated above, deletion of *SCH9* slowed down sugar assimilation, and this effect increased when the mutant was also *petite*, although fermentation was finally completed. Therefore, mitochondria have no major impact on carbon metabolism during grape juice fermentation, but it impacts CLS.

**Fig 7:**
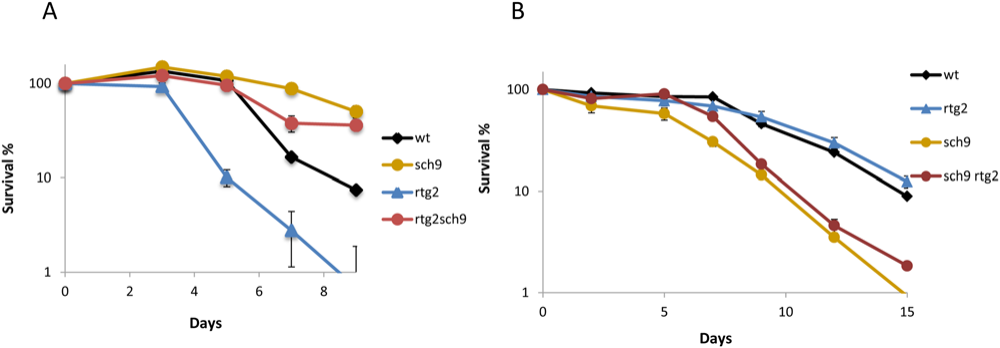
Sch9 is required for CLS regulation through the retrograde response. **A)** Survival curves of the wild type and the single and double *sch9*Δ and *rtg2*Δ mutants in SC medium. **B)** Survival plot of the same strains during grape juice fermentation. Experiments were performed in triplicate. Error bars show the standard deviation (SD).

To further investigate the relationship between the Sch9 kinase and mitochondria, we focussed on the involvement of the retrograde response as it is known that the TOR pathway is a regulator of the retrograde pathway [18]. Mutations in *SCH9* and *RTG2* genes were combined, and the CLS profiles were studied in SC laboratory medium (Fig. 7A) and in grape juice (Fig. 7B). In SC, *SCH9* deletion caused CLS extension, whereas *RTG2* led to CLS shortening, as previously described. The opposite behaviors were observed when CLS was determined in grape juice, where *RTG2* deletion caused CLS extension and *SCH9* deletion led to CLS shortening. In both cases, however, the double *sch9*Δ *rtg2*Δ mutant had an intermediate life span, with higher longevity than the parental strain in SC medium (Fig. 7A) and a shorter one in grape juice (Fig. 7B). These results suggest that the effect of the retrograde response on life span requires Sch9 to be fully channeled. Under winemaking conditions, the situation was reversed. *RTG2* deletion caused CLS extension, which was blocked by the mutation of the *SCH9* gene, that was short-lived compared to the wild-type strain. This confirms the idea that Sch9 is required to transmit information from the mitochondria that carry the retrograde response and to bring about a change in CLS.

## Discussion

The environmental conditions for *S. cerevisiae* growth determine the fate of the yeast population in the death phase. It is well-known that growth in a medium with reduced glucose content, i.e., calorie restriction, extends both CLS and RLS [4,5]. When a nonfermentable carbon source is used, longevity is also extended. The effect of nitrogen source depletion on life span is more complex, probably due to the variety of nitrogen sources that yeasts can use (from ammonia to any of the 20 amino acids). The fact that most life span experiments have been performed with laboratory auxotrophic strains spells complexity due to potential intracellular metabolic unbalances. When we used prototrophic industrial wine strains, which are capable of growing with ammonia or with any of the 20 amino acids as a single nitrogen source, we observed that global nitrogen reduction (by reducing both ammonia and amino acids) extended CLS during winemaking fermentation [9,40]. Starvation of selected amino acids has a complex impact on life span, with some acting as pro-aging factors and others have an anti-aging effect [41–43]. In any case, nutrient sensing pathways are the master regulators of life span extension caused by dietary restriction, and TOR/Sch9 is particularly devoted to the cellular response to the nitrogen composition of growth medium. Wine strains respond to carbon source restriction by showing typical CLS extension [44].

Some cellular mechanisms have an invariable effect on life span, regardless of the growth conditions. Examples of such consistent effects are PKA activity, which has negative effect on life span under both laboratory and winemaking conditions; and the stress response, which is positive for longevity [40]. However, we have already described other processes, accepted as being necessary to achieve full chronological life span under laboratory conditions, and are detrimental to CLS during grape juice fermentation. For instance, deletion of the *ATG7* gene abolishes autophagy and then causes a reduced CLS in laboratory medium, as expected, but extends life span under winemaking conditions, which indicates that autophagy is detrimental for longevity during grape juice fermentation [29]. However, deletion of the *SPT20* gene, and then dismantling the SAGA complex, block autophagy (Fig. 1C), but CLS shortening is observed in the *spt20*Δ mutant in both SC medium (Fig. 1A) and grape juice (Fig. 2B). Given the relevant role of the SAGA complex in other cellular processes, such as stress response [45], it is believed that any effect of autophagy on life span under winemaking conditions can be overcome when SAGA integrity is lost. Given this variety of cellular outcomes, a picture of aging as a very complex trait where many genetic and environmental factors can act together to achieve full life span, as has emerged in recent years.

This work describes some other mutations in the genes that code for life span-controlling proteins, which play opposite roles in different growth conditions and environments, proteins that are involved in distinct cellular regulatory mechanisms. One of these mechanisms is retrograde pathways, which respond to failure in mitochondrial electrochemical potential and send information to the nucleus to activate the transcription of nuclear genes with a mitochondrial function [18,46]. Rtg2 is an essential component of this pathway and its deletion in wine yeasts produces a drop in CLS in SC medium under aeration (Fig. 3A), which is consistent with the need for respiration when sugars in the medium are consumed. However, the same *rtg2*Δ mutant displays an even slightly extended CLS in winemaking, where respiration is not required (Fig. 3B), and the energy waste of expressing respiratory proteins under such conditions may be detrimental for cell survival. However, this simplified view of mitochondrial function does not offer a complete explanation of its interplay with life span as *petite* mutants are incapable of respiration and have a shorter CLS in both SC and also in grape juice (Fig. 6). The behavior of *petite* mutants if compared to the inhibition of the retrograde response by *RTG2* deletion suggests that mitochondrial integrity and/or metabolic roles, other than respiration itself, might be relevant to achieve full life span during grape juice fermentation because it was not affected significantly by loss of mitochondria-nucleus signaling.

The effect of the *SCH9* deletion on CLS in winemaking is a challenge for the accepted role of nutrient sensing pathways in aging (Fig. 5). The Sch9 kinase integrates various pathways to promote growth and protein synthesis, and its deletion extends both CLS and RLS in laboratory media [7,8]. This is also the case of wine yeast when CLS is assayed during growth in SC medium (Fig. 4A), but not during grape juice fermentation, where the *sch9*Δ mutant shows shortened life span (Fig. 5). The specific media composition components causing this difference can be argued, such as the particular high carbon/low nitrogen ratio of natural grape juice, which determines a clearly different dietary restriction. To support this interpretation, *SCH9* deletion also shortened CLS (Fig. 6A) in the reproducing CLS experiments performed in a modified SC with very low total nitrogen. Thus it can be concluded that nutritional unbalances are key factors in the impact of *SCH9* deletion on CLS as a *sch9*Δ mutant extends longevity during synthetic grape juice fermentation with a high nitrogen and low lipid composition, and this situation promotes cell death [47]. It has been suggested that the life span extension caused by Sch9 inhibition may be due to an increased respiratory rate [39]. The effect of *SCH9* deletion on life span during winemaking also seems to be dependent on mitochondrial integrity since a *petite* version of the *sch9*Δ mutant displayed no further decrease in CLS, but reduced the CLS of the wild-type strain (Fig. 6B). In grape juice or SC with low nitrogen, poor amino abundance acids may complicate protein translation, promoted by Sch9. Such shortage in translation may be detrimental when sugars are plenty and respiration is irrelevant. This may explain why double mutant *sch9*Δ *rtg2*Δ more resembles the *sch9*Δ mutant (short-lived) than the *rtg2*Δ mutant (long-lived) under winemaking conditions (Fig. 7B). Finally, a relationship exists between TOR/Sch9 and the SAGA complex as *gcn5*Δ mutation prevents, at least partially, CLS extension in SC medium caused by deletions *SCH9* and *TOR1*. That suggests that the gene expression changes caused by the TOR/Sch9 pathway are channeled, at least partially, through the SAGA complex. During grape juice fermentation, the *gcn5*Δ *sch9*Δ double mutant displayed a shortened CLS like the *sch9*Δ simple mutant, which suggests that the role of SAGA in gene expression is also dependent on growth conditions. *SCH9* and *GCN5* interact with a new life span regulator, transcription factor *RGM1*. Although Rgm1 seems to have no impact on longevity during normal cellular growth, it appears to be important for CLS in starvation, like those mimicked by the *sch9*Δ mutation (Fig. 5). It has been recently established a link between SAGA and TOR pathway. The ribosomal transcription factor Ifh1 is acetylated by Gcn5 and its phosphorylation is mediated by TORC1 to modulate replicative life span [48].

## Supporting Information

S1 Fig Spt20 plays a role in growth and aging under winemaking conditions also in the haploid wine strain C9. **A)** Growth curves for wine strain C9 and its derivatives *spt20*Δ and *ubp8*Δ showing the number of viable cells (cfu/mL) determined by plate counting at different times during winemaking in synthetic grape juice. Experiments were performed at least in triplicate, and errors bars show the standard deviation (SD). **B)** Survival curves for the same strains. The cell numbers at day 3 in panel A were taken as 100% viability. **C)** Sugar consumption profiles during fermentation. **D)** Ethanol production at the end of grape juice fermentation. Ethanol was measured when sugars were completely consumed (below 2 g/l).
(PDF)

S2 Fig Deletion of *RTG2* has no impact of grape juice performance. Sugar consumption profiles during fermentation (A) and ethanol production at the end of grape juice fermentation described in Fig. 3B.
(PDF)

S3 Fig Deletion of *RGM1* has no impact of grape juice performance. Sugar consumption profiles during fermentation (A) and ethanol production at the end of grape juice fermentation described in Fig. 5.
(PDF)

S1 Table Yeast strains used in this work (DOCX)

S2 Table Oligonucleotides used in this work. Oligonucleotide pair a/b were used to amplify the *kanMX*-containing disruption cassette for each gene. Oligonucleotide c hybridizes to the promoter, oligonucleotide d to the coding sequence and oligonucleotide e to the terminator of each gene. Oligonucleotide K2 matches the selection marker *kanMX*. Pair c/K2 would give a PCR product is the selected gene is disrupted. Pair c/d will give a PCR product if a copy of the gene is still present in the cell. Pair c/e would give a small PCR product if the selection marker has been eliminated after recombinase cre induction.
(DOCX)

S3 Table Mean and Maximum CLS for the strains in the figures indicated. Data was obtained with the Prism GraphPad software package.
(DOCX)
