# Supplementary figures and images for: Interplay among Gcn5, Sch9 and Mitochondria during Chronological Aging of Wine Yeast Is Dependent on Growth Conditions
Source: PLoS One. 2015 Feb 6;10(2):e0117267. doi: 10.1371/journal.pone.0117267 (PMC4319768; doi:10.1371/journal.pone.0117267)

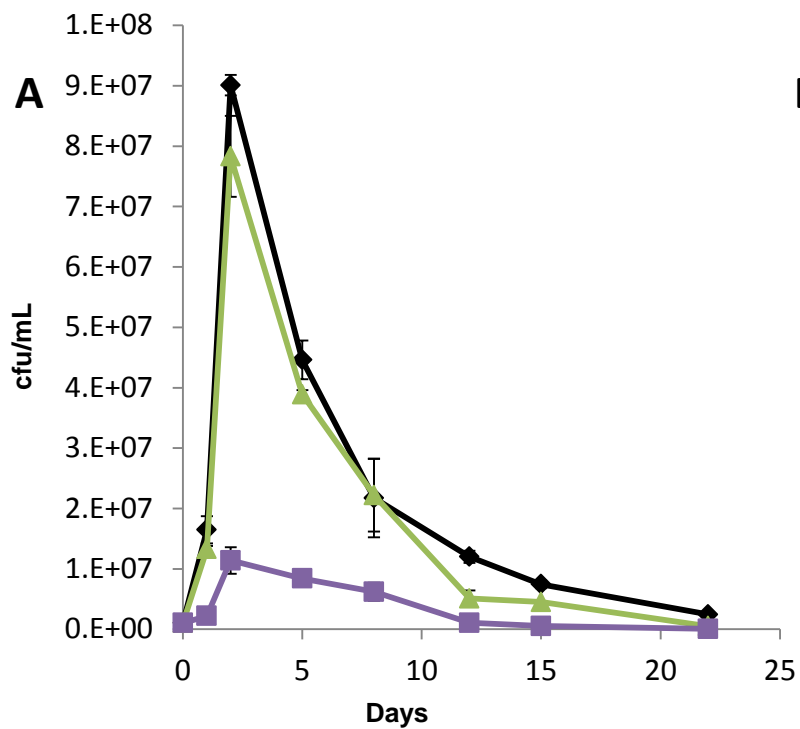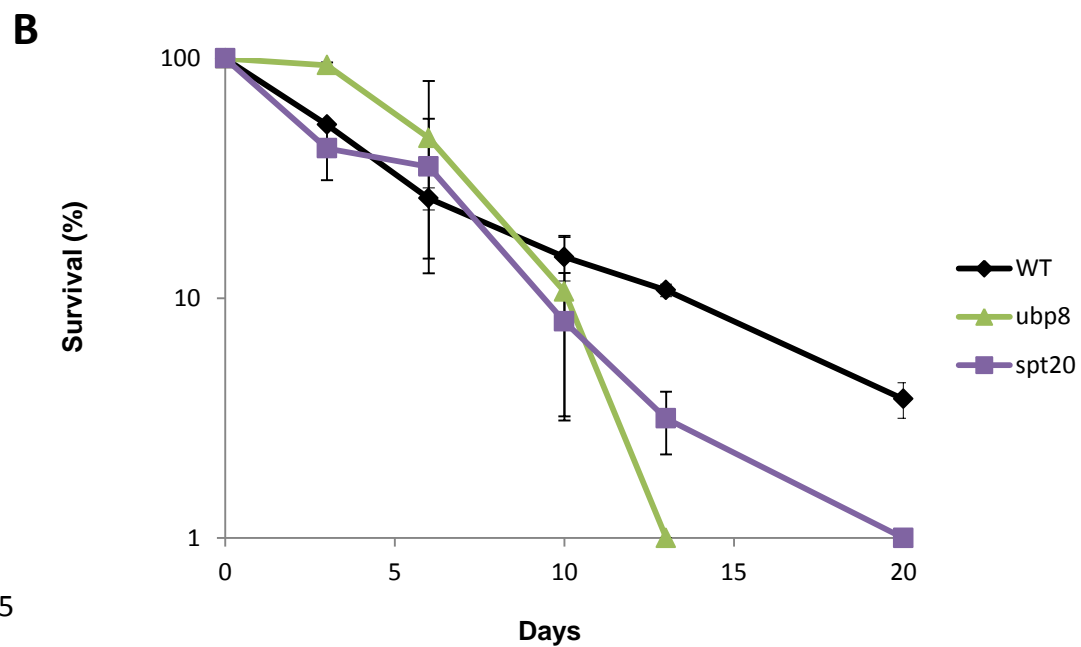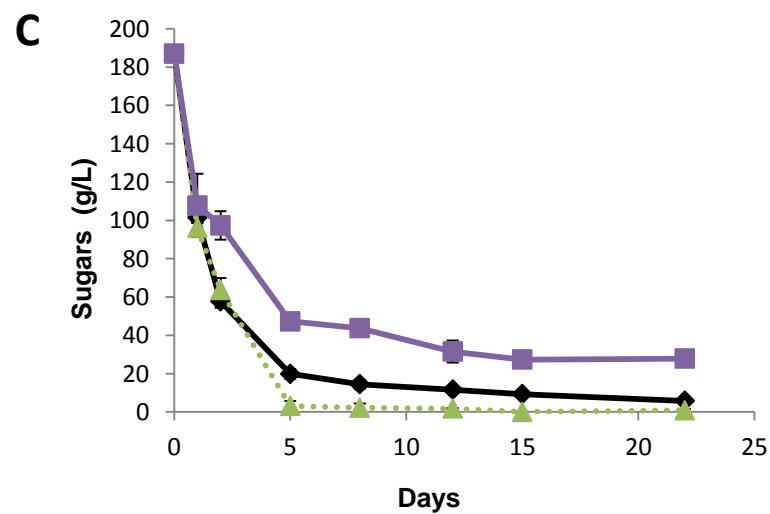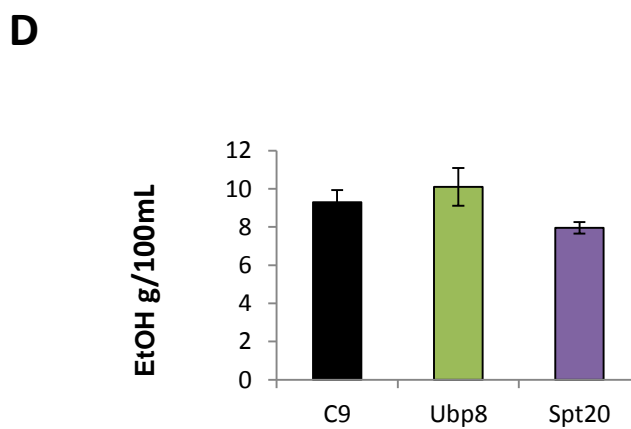

Supplement: S1 Fig — A) Growth curves for wine strain C9 and its derivatives spt20Δ and ubp8Δ showing the number of viable cells (cfu/mL) determined by plate counting at different times during winemaking in synthetic grape juice. Experiments were performed at least in triplicate, and errors bars show the standard deviation (SD). B) Survival curves for the same strains. The cell numbers at day 3 in panel A were taken as 100% viability. C) Sugar consumption profiles during fermentation. D) Ethanol production at the end of grape juice fermentation. Ethanol was measured when sugars were completely consumed (below 2 g/l). (PDF) [file pone.0117267.s001.pdf]

**A)**

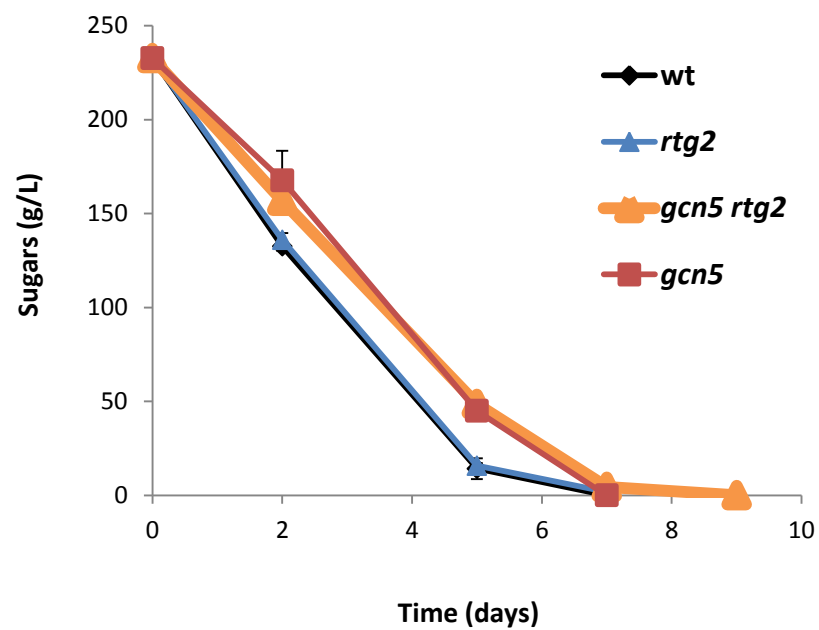

**B)**

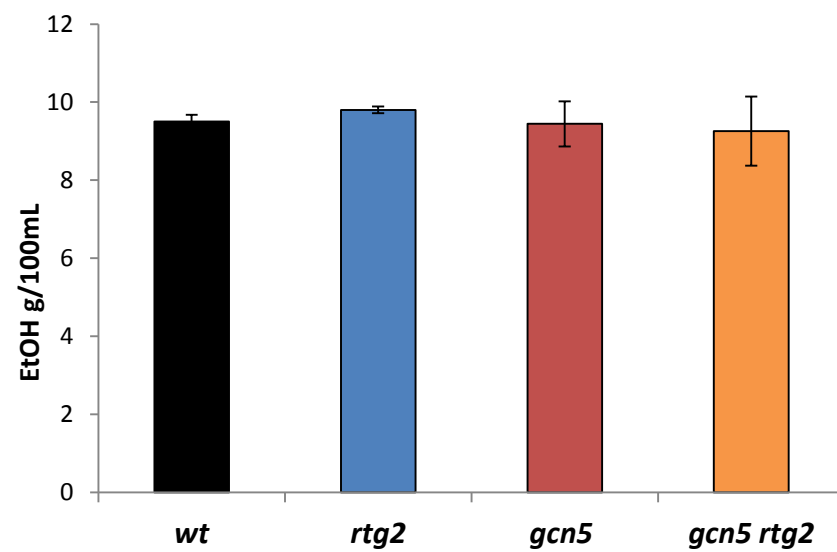

Supplement: S2 Fig — Sugar consumption profiles during fermentation (A) and ethanol production at the end of grape juice fermentation described in Fig. 3B. (PDF) [file pone.0117267.s002.pdf]

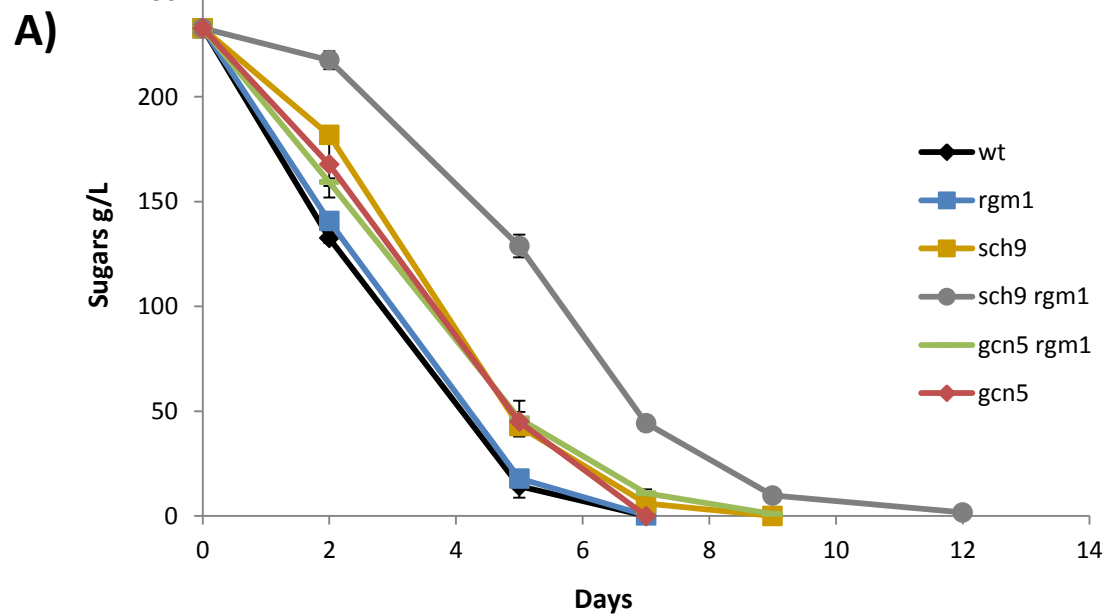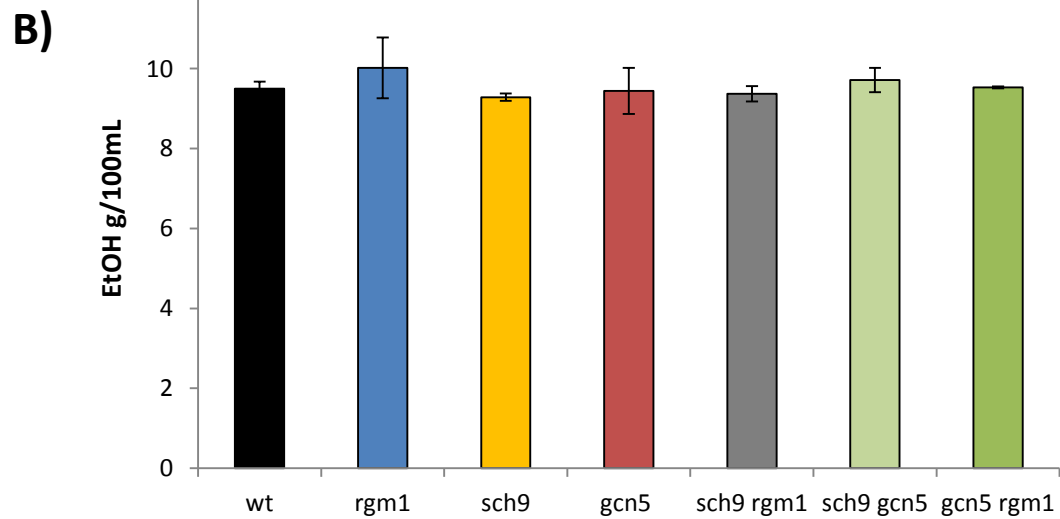

Supplement: S3 Fig — Sugar consumption profiles during fermentation (A) and ethanol production at the end of grape juice fermentation described in Fig. 5. (PDF) [file pone.0117267.s003.pdf]
