# Supplementary material for: Interplay among Gcn5, Sch9 and Mitochondria during Chronological Aging of Wine Yeast Is Dependent on Growth Conditions
Source: PLoS One. 2015 Feb 6;10(2):e0117267. doi: 10.1371/journal.pone.0117267 (PMC4319768; doi:10.1371/journal.pone.0117267)
Supplement: S1 Table — (DOCX) [file pone.0117267.s004.docx]

**Table S1. Yeast strains used in this work**

| STRAINS | GENOTIPE | ORIGIN |
| --- | --- | --- |
| L2056 | **Wine strain** | **Lallemand Inc. (Montreal, Canada)** |
| L2056 *spt20* | **L2056::*spt20::loxP spt20::KanMX*** | **This work** |
| L2056 *ubp8* | **L2056::*ubp8::loxP ubp8::KanMX*** | **“** |
| C9 | **Mat *a ho::loxP*** | **JM Walker** |
| C9 *gcn5* | **C9::*gcn5::KanMX*** | **Helena Orozco** |
| C9 *ubp8* | **C9::*ubp8:.KanMX*** | **This work** |
| C9 *spt20* | **C9::*spt20::KanMX*** | **This work** |
| C9 *ald4* | **C9::*ald4::KanMX*** | **Helena Orozco** |
| C9 *ubp8 ald4* | **C9::*ubp8::loxP ald4:: KanMX*** | **This work** |
| C9 *spt20 ald4* | **C9::*spt20::loxP ald4::KanMX*** | **“** |
| C9 *tor1* | **C9::*tor1::KanMX*** | **Helena Orozco** |
| C9 *sch9* | **C9::*sch9::KanMX*** | **This work** |
| C9 *rtg2* | **C9::*rtg2::KanMX*** | **“** |
| C9 *gcn5* | **C9::*gcn5::loxP*** | **“** |
| C9 *tor1* | **C9::*tor1::loxP*** | **“** |
| C9 *sch9* | **C9::*sch9::loxP*** | **“** |
| C9 *rgm1* | **C9::*rgm1::KanMX*** | **“** |
| C9 *tor1gcn5* | **C9:: *tor1::loxP gcn5::KanMX*** | **“** |
| C9 *sch9gcn5* | **C9::*sch9::loxP gcn5::KanMX*** | **“** |
| C9 *gcn5rtg2* | **C9*::gcn5::loxP rtg2::KanMX*** | **“** |
| C9 *gcn5rgm1* | **C9*::gcn5::loxP rgm1::KanMX*** | **“** |
| C9 *sch9rtg2* | **C9*::sch9::loxP rtg2::KanMX*** | **“** |
| C9 *sch9 rgm1* | **C9*::sch9::loxP rtg2::KanMX*** | **“** |
|  |  |  |
|  |  |  |
|  |  |  |
