# Supplementary material for: Interplay among Gcn5, Sch9 and Mitochondria during Chronological Aging of Wine Yeast Is Dependent on Growth Conditions
Source: PLoS One. 2015 Feb 6;10(2):e0117267. doi: 10.1371/journal.pone.0117267 (PMC4319768; doi:10.1371/journal.pone.0117267)
Supplement: S2 Table — Oligonucleotide pair a/b were used to amplify the kanMX-containing disruption cassette for each gene. Oligonucleotide c hybridizes to the promoter, oligonucleotide d to the coding sequence and oligonucleotide e to the terminator of each gene. Oligonucleotide K2 matches the selection marker kanMX. Pair c/K2 would give a PCR product is the selected gene is disrupted. Pair c/d will give a PCR product if a copy of the gene is still present in the cell. Pair c/e would give a small PCR product if the selection marker has been eliminated after recombinase cre induction. (DOCX) [file pone.0117267.s005.docx]

**Table S2. Oligonucleotides used in this work**

Oligonucleotide pair a/b were used to amplify the *kanMX*-containing disruption cassette for each gene. Oligonucleotide c hybridizes to the promoter, oligonucleotide d to the coding sequence and oligonucleotide e to the terminator of each gene. Oligonucleotide K2 matches the selection marker *kanMX*. Pair c/K2 would give a PCR product is the selected gene is disrupted. Pair c/d will give a PCR product if a copy of the gene is still present in the cell. Pair c/e would give a small PCR product if the selection marker has been eliminated after recombinase cre induction.

| Oligonucleotide | Sequence |
| --- | --- |
| SPT20a | AGGAATAGTTACGGTTAATTTGCGCCTATATATTTCAGGGTTCGTACGCTGCAGGTCGAC |
| SPT20b | TATATATATATATAAGGAATGATAACTCTATTTAAGTAGAATAGGCCACTAGTGGATCTG |
| SPT20c | TTAGTTCTGTTACCCGCTCG |
| SPT20d | TTACATTTGCTACCGCTGGG |
| SPT20e | TTGAGCACAGAGGTGAGATG |
| UBP8a | CTACTTGAAACCCTGCTTTTTTTATTTGTTATTAATAATTTTCGTACGCTGCAGGTCGAC |
| UBP8b | CTTTTTTGTTTTATTATTATTGTTGAATGCTATTTGCTGAATAGGCCACTAGTGGATCTG |
| UBP8c | GCTGTCGTAATAGCAAAGGG |
| UBP8d | GCACGTTTTTAGAACCCCATC |
| UBP8d | CCAGCCTTTGCTCTTTTGTTCG |
| GCN5a | AAAAGTCTTCAGTTAACTCAGGTTCGTATTCTACATTAGTTCGTACGCTGCAGGTCGAC |
| GCN5b | CTTCGAAAGGAATAGTAGCGGAAAAGCTTCTTCTACGCAATAGGCCACTAGTGGATCTG |
| GCN5c | AGGATTGGTAAGGGAAGACC |
| GCN5d | TTATTGGTCTCAGCCTGCTC |
| GCN5e | ATTGATCACATCGTCTCGCC |
| SCH9a | ATACTCGTATAAGCAAGAAATAAAGATACGAATATACAATTTCGTACGCTGCAGGTCGC |
| SCH9b | AAGGAAAAGAAGAGGAAGGGCAAGAGGAGCGATTGAGAAAATAGGCCACTAGTGGATCTG |
| SCH9c | CCCACTCTCACATAATCACC |
| SCH9d | ATGGGTATCCGTTGTCGTTG |
| SCH9e | GCGAAGCGTTTACTTAAGCC |
| TOR1a | AGTGAAACATACATCAACCGGCTAGCAGGTTTGCATTGATTTCGTACGCTGCAGGTCGAC |
| TOR1b | AAATAGTAAACAAAGCACGAAATGAAAAATGACACCGCAGATAGGCCACTAGTGGATCTG |
| TOR1c | ACCTACTAGAAGTCAGACCG |
| TOR1d | GGTTCTCGCCATTTTGAAGC |
| TOR1e | AGATGAGGACCTCAGTAAGG |
| RTG2a | ATGTCAACACTTAGCGATAGTGATACCGAGACTGAGGTCGTTCGTACGCTGCAGGTCGAC |
| RTG2b | TCACATACATAATCGTACCAATATACTTCGTCCACCATACATAGGCCACTAGTGGATCTG |
| RTG2c | AGTCACATGACCGCGATAAG |
| RTG2d | TTCTTGCATGATGTGCAGCC |
| RGM1a | CGTCTATAGTCCAGGACAATTAGCATGACACTGGTTAAACTTCGTACGCTGCAGGTCGAC |
| RGM1b | TGATGCGGCTTGTGAAATGGAGGAGTGGGTGCGGTAGAGCATAGGCCACTAGTGGATCTG |
| RGM1c | TTCATTCCTCAGCGGATCCC |
| RGM1e | CATTGGAATGGTTTCTCCCC |
| K2 | GGGACAATTCAACGCGTCTG |
