# Supplementary material for: Interplay among Gcn5, Sch9 and Mitochondria during Chronological Aging of Wine Yeast Is Dependent on Growth Conditions
Source: PLoS One. 2015 Feb 6;10(2):e0117267. doi: 10.1371/journal.pone.0117267 (PMC4319768; doi:10.1371/journal.pone.0117267)
Supplement: S3 Table — Data was obtained with the Prism GraphPad software package. (DOCX) [file pone.0117267.s006.docx]

| Table S3. Mean and Maximum CLS for the strains in the figures indicated.  Data was obtained with the Prism GraphPad software package. | | | | | | |  |  |  | | |  |  |  |
| --- | --- | --- | --- | --- | --- | --- | --- | --- | --- | --- | --- | --- | --- | --- |
| Figure | Strain | Mean CLS (50%Viability) | | | Maximum CLS (10%viability) | | |  |  |  |  |  |  |  |
| 1A | wt | 4,64 | | | 14,335 | | |  |  |  |  |  |  |  |
|  | *gcn5* | 3,275 | | | 7,958 | | |  |  |  |  |  |  |  |
|  | *ubp8* | 5,683 | | | 15,9 | | |  |  |  |  |  |  |  |
|  | *spt20* | 3,475 | | | 6,953 | | |  |  |  |  |  |  |  |
|  |  |  | | |  | | |  |  |  |  |  |  |  |
|  |  |  | | |  | | |  |  |  |  |  |  |  |
| 1B | wt | 11,681 | | | >17 | | |  |  |  |  |  |  |  |
|  | *gcn5* | 6,969 | | | 8,924 | | |  |  |  |  |  |  |  |
|  | *ubp8* | 11,956 | | | >17 | | |  |  |  |  |  |  |  |
|  | *spt20* | 2,994 | | | 9,958 | | |  |  |  |  |  |  |  |
|  |  |  | | |  | | |  |  |  |  |  |  |  |
|  |  |  | | |  | | |  |  |  |  |  |  |  |
| 2A | wt | 16,793 | | | 21,972 | | |  |  |  |  |  |  |  |
|  | *ubp8* | 14,231 | | | 17,909 | | |  |  |  |  |  |  |  |
|  | *spt20* | 8,01 | | | 10,025 | | |  |  |  |  |  |  |  |
|  |  |  | | |  | | |  |  |  |  |  |  |  |
|  |  |  | | |  | | |  |  |  |  |  |  |  |
| 3A | wt | 9,686 | | | 15,581 | | |  |  |  |  |  |  |  |
|  | *gcn5* | 7,385 | | | 13,627 | | |  |  |  |  |  |  |  |
|  | *rtg2* | 5,564 | | | 6,508 | | |  |  |  |  |  |  |  |
|  | *gcn5rtg2* | 2,752 | | | 4,603 | | |  |  |  |  |  |  |  |
|  |  |  | | |  | | |  |  |  |  |  |  |  |
|  |  |  | | |  | | |  |  |  |  |  |  |  |
| 3B | wt | 9,198 | | | 14,417 | | |  |  |  |  |  |  |  |
|  | *gcn5* | 9,9 | | | 18,709 | | |  |  |  |  |  |  |  |
|  | *rtg2* | 9,25 | | | 16,655 | | |  |  |  |  |  |  |  |
|  | *gcn5rtg2* | 14,508 | | | 27,367 | | |  |  |  |  |  |  |  |
| Figure | **Strain** | **Mean CLS (50%Viability)** | | | **Maximum CLS (10%viability)** | | |  | | | **Maximum CLS (10%viability)** | | |  |
| 4A | wt | 1.923 | | | 3.316 | | |  |  |  |  |  |  |  |
|  | *gcn5* | 0.484 | | | 1.603 | | |  |  |  |  |  |  |  |
|  | *sch9* | >8 | | | >8 | | |  |  |  |  |  |  |  |
|  | *sch9gcn5* | 3.207 | | | 4.965 | | |  |  |  |  |  |  |  |
|  |  |  | | |  | | |  |  |  |  |  |  |  |
|  |  |  | | |  | | |  |  |  |  |  |  |  |
| 4B | *wt* | 3.57 | | | 6.746 | | |  |  |  |  |  |  |  |
|  | *gcn5* | 1.5 | | | 2.69 | | |  |  |  |  |  |  |  |
|  | *tor1* | 3.247 | | | >8 | | |  |  |  |  |  |  |  |
|  | tor1gcn5 | 1.674 | | | 2.132 | | |  |  |  |  |  |  |  |
|  |  |  | | |  | | |  |  |  |  |  |  |  |
|  |  |  | | |  | | |  |  |  |  |  |  |  |
| 4C | *wt* | 9.686 | | | 15.581 | | |  |  |  |  |  |  |  |
|  | *gcn5* | 7.385 | | | 13.627 | | |  |  |  |  |  |  |  |
|  | *sch9* | 10.349 | | | 25.36 | | |  |  |  |  |  |  |  |
|  | rgm1 | 10.907 | | | 15.306 | | |  |  |  |  |  |  |  |
|  | *gcn5rgm1* | 7.9 | | | 14.674 | | |  |  |  |  |  |  |  |
|  | *sch9rgm1* | 8.617 | | | 18.596 | | |  |  |  |  |  |  |  |
|  |  |  | | |  | | |  |  |  |  |  |  |  |
| 5B | *wt* | 9.198 | | | 14.417 | | |  |  |  |  |  |  |  |
|  | *rgm1* | 8.333 | | | 13.187 | | |  |  |  |  |  |  |  |
|  | *sch9* | 4.914 | | | 10.697 | | |  |  |  |  |  |  |  |
|  | *gcn5* | 9.9 | | | 18.709 | | |  |  |  |  |  |  |  |
|  | *sch9rgm1* | 9.218 | | | 11.771 | | |  |  |  |  |  |  |  |
|  | gcn5rgm1 | 12.691 | | | 22.451 | | |  |  |  |  |  |  |  |
|  |  |  | | |  | | |  |  |  |  |  |  |  |
|  |  |  | | |  | | |  |  |  |  |  |  |  |
| Figure | **Strain** | **Mean CLS (50%Viability)** | | | **Maximum CLS (10%viability)** | | |  |  |  |  |  |  |  |
| 6A | wt | 19.145 | | |  | | |  |  |  |  |  |  |  |
|  | *sch9* | 6.823 | | |  | | |  |  |  |  |  |  |  |
|  |  |  | | |  | | |  |  |  |  |  |  |  |
| 6B | *wt* | 4.04 | |  | | | |  | | | | |  |  |
|  | *sch9* | >9 | |  | | | |  | | | | |  |  |
|  | *wt rho* | 2.973 | | 5.144 | | | |  | | | | |  |  |
|  | *sch9rho* | 2.982 | | 5.705 | | | |  | | | | |  |  |
|  |  |  | |  | | | |  |  |  |  |  |  |  |
| 6C | *wt* | 11.237 | | 20.638 | | | |  | | | | | 15.581 | |
|  | *sch9* | 8.696 | | 13.094 | | | |  | | | | | 13.627 | |
|  | *wt rho* | 9.128 | | 12.541 | | | |  | | | | | 25.36 | |
|  | *sch9rho* | 8.834 | | 13.544 | | | |  | | | | | 15.306 | |
|  |  |  | |  | | | |  |  |  |  |  |  |  |
| 7A | *wt* | 6.171 | | 6.839 | | | |  |  |  |  |  |  |  |
|  | *rtg2* | 4.051 | | 4.637 | | | |  |  |  |  |  |  |  |
|  | *sch9* | >8 | | >8 | | | |  |  |  |  |  |  |  |
|  | *sch9rtg2* | 5.87 | | >8 | | | |  |  |  |  |  |  |  |
|  |  |  | |  | | | |  |  |  |  |  |  |  |
| 7C | *wt* | 9.198 | | 14.417 | | | |  |  |  |  |  |  |  |
|  | *rtg2* | 9.25 | | 16.655 | | | |  |  |  |  |  |  |  |
|  | *sch9* | 4.914 | | 10.697 | | | |  |  |  |  |  |  |  |
|  | *sch9rtg2* | 7.294 | | 9.667 | | | |  |  |  |  |  |  |  |
|  |  | |  | | |  | | | |  |  |  |  |  |
